# Evaluating the Lidocaine's Initial Dosing in Patients With Ventricular Arrhythmias and Heart Failure Admitted in Intensive Care Units

**DOI:** 10.1002/clc.70126

**Published:** 2025-04-03

**Authors:** Kazuhiko Kido

**Affiliations:** ^1^ Department of Clinical Pharmacy School of Pharmacy West Virginia University Morgantown West Virginia USA

**Keywords:** cardiogenic shock, heart failure, lidocaine, pharmacokinetics, therapeutic drug monitoring

## Abstract

**Introduction:**

Lidocaine is an antiarrhythmic with narrow therapeutic window indicated for refractory ventricular arrhythmia. Limited guidance is available regarding the initial infusion rate ranging from 1 to 4 mg/min in patients with heart failure (HF).

**Objectives:**

The primary objective was to assess the optimal initial dosing of lidocaine therapy in patients with HF and ventricular arrhythmia.

**Methods:**

The retrospective cohort study was performed to include patients aged 18 years or older with past medical history of HF or new onset HF who developed ventricular arrythmia requiring lidocaine therapy in cardiovascular intensive care units. The primary outcome was lidocaine levels within the therapeutic ranges (defined as 1.5 to 5.0 μg/L). The study also described the predictive performance of lidocaine one compartment PK model using correlation efficient between the population PK model‐predicted levels and observed levels.

**Results:**

A total of 56 patients with HF and ventricular arrhythmia was included. The mean lidocaine maintenance dose was 1.1 ± 0.5 mg/min. The median (IQR) lidocaine level was 3.1 (2.3, 4.1) μg/L. The probabilities within therapeutic, supratherapeutic, and subtherapeutic ranges were 66.1%, 19.6%, and 14.3%, respectively in the whole cohort. Predicted lidocaine levels with one compartment PK model were not correlated with observed lidocaine levels (*R* ^2^ = 0.34). The simulation investigation showed that 1 mg/min rate achieved the highest probability within therapeutic range compared to 0.5, 1.5, and 2.0 mg/min rates (78.6 vs. 53.6, 53.6, and 28.6%, respectively).

**Conclusion:**

Lidocaine initial infusion rate should be up to 1 mg/min in patients with HF and ventricular arrhythmia.

## 1 Introduction

According to the American College of Cardiology/American Heart Association/Heart Rhythm Society guidelines for ventricular arrhythmia, lidocaine is an antiarrhythmic indicated mainly for shock refractory ventricular tachycardia (VT)/ventricular fibrillation, especially in the setting of acute myocardial infarction [1]. Lidocaine requires close therapeutic drug monitoring (TDM) to maintain the levels within the therapeutic range of 1.5–5.0 μg/L and monitor efficacy and adverse drug effects [2]. If the level is above 5.0 μg/L, lidocaine toxicities such as muscle twitching, agitation, dysarthria, psychosis, seizure, coma, and confusion may occur [3]. Thus, lidocaine levels are measured at steady‐state (4–5 half‐lives) if the therapy is continued for a longer duration (e.g., > 24 h) or if patients experience recurrence of ventricular arrhythmia or lidocaine toxicities. Significant delays in lidocaine level results may prolong the duration of inappropriately lower or higher lidocaine dosing or potentially overlook accumulation of lidocaine for patients needing a longer duration of lidocaine therapy > 24 h. Since lidocaine is dominantly eliminated by liver metabolism via CYP3A (> 95%) and its clearance is liver blood flow related, any diseases decreasing liver flow, such heart failure or especially cardiogenic shock, can decrease lidocaine clearance [4, 5]. Thus, patients with HF or presenting with cardiogenic shock should be monitored with more frequent lidocaine levels [4, 6]. Although lidocaine PK was recently reviewed in general populations, including lidocaine for pain control, limited data comparing predictive performance in different initial dosing methods is available to select the appropriate initial dose in patients with HF and ventricular arrhythmia [7]. The lidocaine initial dosing is currently selected with a wide range of infusion rates from 1 to 4 mg/min regardless of comorbidities, weight, and organ function. The primary objective of the study was to assess the optimal initial dosing of lidocaine therapy in patients with HF and ventricular arrhythmia. The study evaluated the predictive performance of the currently recommended labeling initial dosing with 1 mg/min in patients with HF and ventricular arrhythmia. The study also evaluated the predictive performance of the lidocaine pharmacokinetic dosing methods with a one‐compartment pharmacokinetic model in the external cohort. The secondary objective was to describe the current landscape of lidocaine therapy.

## 2 Methods

The retrospective cohort study was performed to include adult patients aged 18 years or older with a past medical history of HF or new‐onset HF who developed ventricular arrythmia requiring lidocaine therapy during hospitalization in intensive care units from January 1, 2020 until January 31, 2024. Patients required at least one lidocaine level drawn on a steady state (defined as at least four half‐lives). Exclusion criteria were patients who had lidocaine levels drawn in a non‐steady state. The primary outcome was the probability of lidocaine levels within the therapeutic ranges (defined as 1.5–5.0 μg/L). The first lidocaine level on a steady state was used for this analysis. The study also described the predictive performance of the lidocaine one‐compartment PK model in the literature [3]. Predicted half‐lives based on comorbidities were 4 h in acute myocardial infarction, 2 h for heart failure, 5 h for patients with liver dysfunction based on Child‐Pugh > 8 scores, and 1–1.5 h for patients without afore‐discussed comorbidities. The elimination factor (Ke) was calculated with predicted half‐lives based on the equation: half‐life/0.693. The predicted volume of distribution (Varea: L/kg) was 1.5 L/kg in adult patients with normal liver function, 2.6 L/kg in liver cirrhosis or acute hepatitis, 1.0 L/kg in heart failure, and 1.5 L/kg in post‐MI. Ideal body weight was used for obese patients > 30% over ideal body weight. Correlation between the population PK model‐predicted and observed levels was evaluated with correlation efficiency. The model performance was evaluated in three following parameters [8]:

– Prediction error (PE) (%) = 100%X(Cpred−Cobs)/Cobs
– Median prediction error (MDPE) for bias = median (PE of all individual patients)
– Median absolute prediction error (MADPE) for inaccuracy = median (absolute number of PE for all individual patients)

MDPE ± 20% and MADPE ± 30% indicate an unbiased and accurate model. The percentage of PE falling within ± 20% and ± 30% were estimated as F20 and F30, respectively.

The performance of lidocaine infusion rates 0.5, 1.0, 1.5, and 2.0 mg/min was simulated using the assumption of lidocaine linear pharmacokinetics as a probability of levels within the therapeutic range in the cohort with HF and ventricular arrhythmia. The local Institutional Review Board approved the study.

## 3 Results

A total of 56 patients with HF and ventricular arrhythmia was included in the study (Table 1). The most common lidocaine indication was monomorphic VT (53.6%), followed by polymorphic VT (26.8%). Most included patients had HFrEF (76.8%), and the average EF was 30.8% ± 15.2%. Amiodarone was concomitantly administered in 96.4% of the cohort. About 64% had acute coronary syndrome, and only 3.6% had cirrhosis.

**Table 1:** Baseline characteristics of lidocaine therapy in patients with heart failure.

	*N* (%), mean ± SD or median (IQR) *n* = 56
Age (year)	63.9 ± 13.8
Gender (M)	43 (76.8%)
Weight (kg)	96.0 ± 30.3
BMI (kg/m^2^)	31.0 ± 8.2
Ethnicity	
White	53 (94.6%)
Black	2 (3.6%)
Hispanic	1 (1.8%)
Asian	0
Indication for lidocaine	
Polymorphic VT	15 (26.8%)
Monomorphic VT	30 (53.6%)
Ventricular fibrillation	9 (16.0%)
Pulseless VT	2 (3.6%)
Mechanical circulatory support	
None	42 (75.0%)
Intra‐aortic balloon pump	4 (7.1%)
VA‐ECMO	2 (3.6%)
Percutaneous temporal left ventricular assist device	8 (14.3%)
HF type	
HFrEF	43 (76.8%)
HFmrEF	6 (10.7%)
HFpEF	7 (12.5%)
EF (%)	30.8 ± 15.2
GDMT use at home (%)	
Beta‐blocker	35 (62.5%)
Sacubitril/valsartan	7 (12.5%)
Mineralocorticoid antagonist	8 (14.3%)
SGLT 2 inhibitor	6 (10.7%)
Amiodarone when lidocaine was started	54 (96.4%)
AST (U/L)	43 (29, 100)
ALT (U/L)	39 (20, 76)
Total bilirubin (mg/dL)	0.7 (0.5, 1.4)
Serum albumin (g/dL)	2.8 (2.4, 3.2)
INR	1.23 (1.08, 1.46)
Serum creatinine (mg/dL)	1.17 (0.93, 1.62)
Cirrhosis	2 (3.6%)
Acute coronary syndrome	36 (64.3%)
Dialysis	6 (10.7%)

Abbreviations: ALT, alanine transaminase; AST, aspartate aminotransferase; EF, ejection fraction; GDMT, guideline directed medical therapy; HF, heart failure; INR, international normalized ratio; VT, ventricular tachycardia.

The details of lidocaine TDM are summarized in Table 2. More than 90% of patients received a lidocaine loading dose at the average dose of 1.1 ± 0.3 mg/kg. The mean lidocaine maintenance dose was 1.1 ± 0.5 mg/min. The median (IQR) lidocaine level was 3.1 μg/L (2.3, 4.1) drawn at the median time of 19.4 h (11.6, 29.6) since initiation of lidocaine infusion. The probabilities within therapeutic, supratherapeutic, and subtherapeutic ranges were 66.1%, 19.6%, and 14.3%, respectively.

**Table 2:** Summary of lidocaine therapy therapeutic drug monitoring.

	*N* (%), mean ± SD or median (IQR), *n* = 56
Loading dose given	51 (91.1%)
Loading dose (mg)	99.5 ± 33.3
Weighted‐based loading dose (mg/kg)	1.1 ± 0.3
Lidocaine drip rate (mg/min)	1.1 ± 0.5
Lidocaine level (µg/mL)	3.1 (2.3, 4.1)
Within therapeutic range (1.5–5)	37 (66.1%)
Supratherapeutic range (5 <)	11 (19.6%)
Subtherapeutic range (< 1.5)	8 (14.3%)
How long did it take to draw the level since drip started (h)	19.4 (11.6, 29.6)
How long did it take to get results (h)	
Whole cohort	38.5 (1.0, 67.6)
In‐house lab	0.9 (0.8, 1.3)
Sent‐out lab	65.7 (54.0, 83.4)
Adverse drug reaction	4 (7.1%): 1 agitation, 1 blurred vision, 1 nausea and vomiting, 1 not specified

Abbreviations: IQR, interquartile; SD, standard deviation.

**Figure 1:**
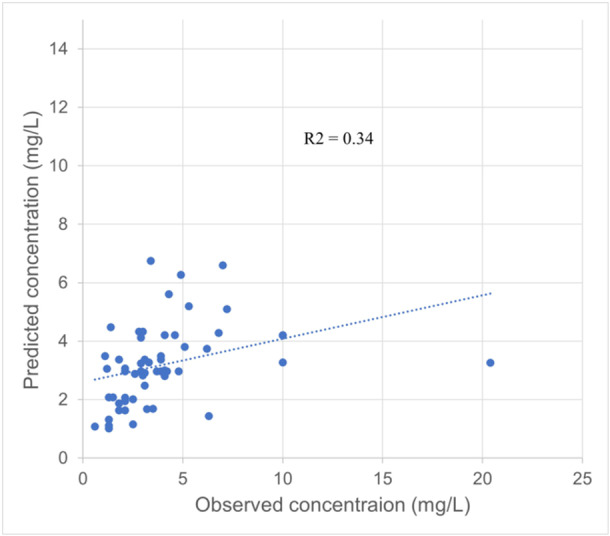
Population pharmacokinetic‐based predicted levels vs. observed lidocaine levels.

One compartment pharmacokinetic model was shown as unbiased and accurate based on MDPE −6.7% (± 20%) and MADPE 28.7% (≤ 30%). F20 = 38.2% (> 30%) and F30 = 56.4% (> 45%) values also met the criteria for sufficient predictive performance of the population pharmacokinetic model in the cohort. The correlation between predicted lidocaine and observed levels was low (*R* ^2 ^= 0.34), and the model appeared to underpredict levels (Figure 1).

**Figure 2:**
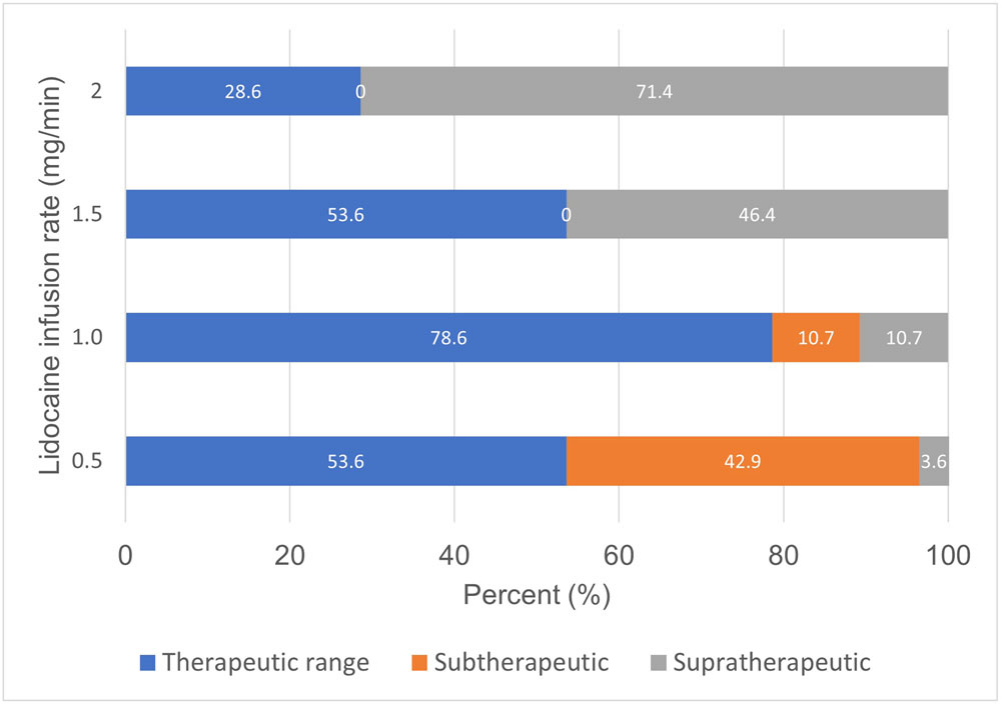
Lidocaine linear pharmacokinetic dosing predictive performance.

A simulation test showed that linear pharmacokinetic dosing with 1.0 mg/min estimated the median lidocaine level (IQR) at 3.1 μg/L (2.4, 4.2). The probabilities within therapeutic, supratherapeutic, and subtherapeutic ranges in patients receiving lidocaine 1 mg/min were 78.6%, 10.7%, and 10.7%, and the probability within the therapeutic range was higher compared to other infusion rates 0.5, 1.5, and 2.0 mg/min (53.6, 53.6, and 28.6%) (Figure 2).

Four adverse drug events (7.1%) were reported (one episode of agitation, one blurred vision, one nausea and vomiting, and one not specified event). Blurred vision and a non‐specified adverse drug event occurred when the levels were 6.3 and 6.8 μg/L, respectively. Nausea and vomiting and agitation occurred when the levels were within the therapeutic range at 4.1 and 3.5 μg/L, respectively.

## 4 Discussion

Our lidocaine pharmacokinetic study showed that lidocaine infusion at 1 mg/min provided probabilities within the therapeutic range at 78.6%, supratherapeutic at 10.7%, and subtherapeutic at 10.7%. However, the probabilities within therapeutic, supratherapeutic, and subtherapeutic ranges in patients receiving lidocaine 0.5 mg/min were only at 53.6% and subtherapeutic at 42.9%. One compartment pharmacokinetic dosing model in the literature was unbiased and accurate and showed acceptable predictive performance. However, the correlation between the predicted and observed levels was low. Our cohort study showed that the mean lidocaine maintenance dose was 1.1 ± 0.5 mg/min, and the median (IQR) lidocaine level was 3.1 μg/L (2.3, 4.1). The cohort's probabilities within therapeutic, supratherapeutic, and subtherapeutic ranges were 66.1%, 19.6%, and 14.3%, respectively.

The experts suggest that lidocaine's initial dosing in patients with HF or liver disease is 1–2 mg/min, but there was a paucity of evidence to support this dosing [3]. Our simulation study with linear pharmacokinetic method showed that the lidocaine infusion rate at 1.0 mg/min achieved the highest probability of therapeutic ranges at 78.6% compared to infusion rates at 0.5, 1.5, and 2.0 mg/min. The initial infusion rate at 2 mg/min caused a supratherapeutic range of 71.4%. Thus, the author recommends dosing up to 1 mg/min in patients with HF hospitalized in intensive care units. The population pharmacokinetic dosing method did not perform well (*R* ^2^ = 0.34) because of lidocaine's narrow therapeutic window and high intervariability in lidocaine clearance due to fluctuated liver blood flow among acutely ill patients with HF.

About 20% or 14% of patients maintained supratherapeutic or subtherapeutic levels in our cohort study, respectively. Three significant variables affecting lidocaine clearance are ACS, HF, and liver disease. About 64% of patients had acute coronary syndrome on admission, which significantly affects lidocaine pharmacokinetic parameters. ACS increased alpha1‐acid glycoprotein by up to 50% over 3 days [6, 9, 10]. The increase in alpha1‐acid glycoprotein decreases lidocaine clearance due to a decreased unbound fraction of lidocaine [3]. Alpha1‐acid glycoprotein continues to increase over time, and lidocaine levels accumulate on extended lidocaine infusion (> 24 h). The status of ACS may increase and accumulate lidocaine levels. Liver cirrhosis or other liver diseases significantly reduce lidocaine clearance because more than 95% of lidocaine is metabolized via the liver [6, 11]. Lastly, patients with HF, especially cardiogenic shock, have reduced lidocaine clearance due to lower cardiac output [4, 6]. ACS, liver disease, and HF necessitate close drug monitoring, especially when patients stay on lidocaine longer than 24 h.

### 4.1 Limitations

Our study has several limitations. First, all the patients in our study had a history of HF or new‐onset HF. Thus, the findings may not apply to patients who do not have HF. Second, the study did not have enough sample size to evaluate the effect of severity of cardiogenic shock and mechanical circulatory support, which significantly affect liver blood flow and lidocaine clearance. Third, this study evaluated the lidocaine initial dosing in the cohort using the initial lidocaine levels. However, lidocaine long‐term therapy was not evaluated for accumulation of lidocaine. Fourth, the study did not have access to the efficacy outcome of ventricular arrhythmia suppression rate. Lastly, our study needs a larger sample size to evaluate variables causing sub‐ and supratherapeutic levels.

## 5 Conclusion

Lidocaine's initial infusion rate should be up to 1 mg/min in patients with heart failure and ventricular arrhythmia. 0.5 mg/min may be considered for patients with higher risks of supratherapeutic levels, such as cardiogenic shock without mechanical circulatory support until cardiac output is restored. One compartment pharmacokinetic model may not be considered to predict the lidocaine levels due to low correlation with observed levels. About 20% had either supra‐ or subtherapeutic levels in the cohort, and about 7% of patients had adverse drug effects. In‐house lidocaine level measurements may be considered in patients with ACS, liver disease, and heart failure who developed ventricular arrhythmia.

## Conflicts of Interest

KazuhikoKido is an independent contractor as a topic editor for DynaMed LLC.

## Data Availability

The data that support the findings of this study are available from the corresponding author upon reasonable request.
